# *S*-Nitrosylation of Surfactant Protein-D Controls Inflammatory Function

**DOI:** 10.1371/journal.pbio.0060266

**Published:** 2008-11-11

**Authors:** Chang-Jiang Guo, Elena N Atochina-Vasserman, Elena Abramova, Joseph P Foley, Aisha Zaman, Erika Crouch, Michael F Beers, Rashmin C Savani, Andrew J Gow

**Affiliations:** 1 Department of Pharmacology and Toxicology, Ernest Mario School of Pharmacy, Rutgers, The State University of New Jersey, Piscataway, New Jersey, United States of America; 2 Division of Pulmonary and Critical Care Medicine, University of Pennsylvania School of Medicine, Philadelphia, Pennsylvania, United States of America; 3 Division of Neonatology, Department of Pediatrics, Joseph Stokes Jr. Research Institute of The Children's Hospital of Philadelphia, Philadelphia, Pennsylvania, United States of America; 4 Department of Pathology and Immunology, Washington University, St. Louis, Missouri, United States of America; 5 Division of Neonatal-Perinatal Medicine, Division of Pulmonary and Vascular Biology, University of Texas Southwestern at Dallas, Dallas, Texas, United States of America; Duke University Medical Center/Howard Hughes Medical Institute, United States of America

## Abstract

The pulmonary collectins, surfactant proteins A and D (SP-A and SP-D) have been implicated in the regulation of the innate immune system within the lung. In particular, SP-D appears to have both pro- and anti-inflammatory signaling functions. At present, the molecular mechanisms involved in switching between these functions remain unclear. SP-D differs in its quaternary structure from SP-A and the other members of the collectin family, such as C1q, in that it forms large multimers held together by the N-terminal domain, rather than aligning the triple helix domains in the traditional “bunch of flowers” arrangement. There are two cysteine residues within the hydrophobic N terminus of SP-D that are critical for multimer assembly and have been proposed to be involved in stabilizing disulfide bonds. Here we show that these cysteines exist within the reduced state in dodecameric SP-D and form a specific target for *S*-nitrosylation both in vitro and by endogenous, pulmonary derived nitric oxide (NO) within a rodent acute lung injury model. *S*-nitrosylation is becoming increasingly recognized as an important post-translational modification with signaling consequences. The formation of *S-*nitrosothiol (SNO)-SP-D both in vivo and in vitro results in a disruption of SP-D multimers such that trimers become evident. SNO-SP-D but not SP-D, either dodecameric or trimeric, is chemoattractive for macrophages and induces p38 MAPK phosphorylation. The signaling capacity of SNO-SP-D appears to be mediated by binding to calreticulin/CD91. We propose that NO controls the dichotomous nature of this pulmonary collectin and that posttranslational modification by *S*-nitrosylation causes quaternary structural alterations in SP-D, causing it to switch its inflammatory signaling role. This represents new insight into both the regulation of protein function by *S*-nitrosylation and NO's role in innate immunity.

## Author Summary

Cells of the lung lining secrete a microbe-binding molecule called surfactant protein D (SP-D) that helps activate the inflammatory system against invading pathogens. In the absence of infection, SP-D is important in limiting inflammation, demonstrated by the fact that mice lacking the SP-D gene have chronic inflammation and emphysema. SP-D has two structural features—a lectin-like head domain and a collagenous tail domain—that, respectively, inhibit and stimulate inflammation. Here we define a mechanism for generating the active “inflammatory” version of SP-D. SP-D is held together in its multimeric state by interacting cysteine residues, which are susceptible to modification by the gaseous second messenger, nitric oxide, to form *S*-nitrosothiols. In this multimeric state, the tail domains are buried, limiting the ability of SP-D to activate inflammation. *S*-nitrosylation causes the multimers to fall apart into trimers, exposing the tail domain. *S*-nitrosylated SP-D induces inflammatory cell activation as determined by chemotaxis, calcium influx, and phosphorylation state. This activity is dependent upon both the *S*-nitrosothiol and the disruption of SP-D's multimeric structure. These modifications are observed in an in vivo model of inflammation and form a critical part of the process. A model is proposed in which nitric oxide operates as a molecular switch for SP-D.

## Abstract 3

Nitric oxide is shown to control the pro- and anti-inflammatory functions of surfactant protein D by altering its quaternary structure via*S*-nitrosylation.

## Introduction

Nitric oxide (NO) has long presented a curious dichotomy within biologic systems, namely that it is both an important physiological regulator and the mediator of many pathologies [1–3]. Nowhere is this more clearly demonstrated than within the pulmonary system. Whereas NO is required for the control of lung vessel and airway dilation, immune defense, and the maintenance of barrier function, it is also a key mediator of acute lung injury, bronchopulmonary dysplasia, respiratory distress syndrome, and asthma [4]. The contradictory behavior of NO is highlighted by the successes and failures of inhaled NO, which has revolutionized the treatment of persistent pulmonary hypertension of the newborn and, potentially, bronchopulmonary dysplasia [5,6], but has failed to help—and may even harm—patients with acute respiratory distress syndrome [7].

The role of NO in signal transduction pathways, other than its activation of the cyclic-GMP (cGMP) pathway [8,9], has become of increasing relevance in recent years. Of particular interest is the capability of NO to induce signaling functions via the post-translational modification of proteins [10–12]. Among these modifications, *S*-nitrosylation is emerging as an important regulator of protein function [13,14]. *S*-nitrosylation is a covalent modification of thiol groups by formation of a thionitrite *S-*nitrosothiol (SNO) group, and can occur either through direct reaction, metal catalysis, or via the formation of higher nitrogen oxides [12]. Over the past decade, more than a hundred proteins [15] have been shown to become *S*-nitrosylated, and in many cases, this modification is accompanied by altered function. The potential for SNO as a post-translational regulator of protein function has been highlighted by recent proteomic and targeted studies [16–18].

In parallel to the dichotomous nature of NO in the lung, the pulmonary collectin surfactant protein D (SP-D) (Swissprot (http://www.ebi.ac.uk/swissprot/index.html) accession number P50404) has been shown to be important in mediating pathogen clearance and the pulmonary inflammatory response, whereas animals lacking SP-D develop spontaneous inflammation within the lung and associated pulmonary disease [19,20]. How the pro- and anti-inflammatory functions of SP-D are regulated is unknown. However, it has been suggested that pro-inflammatory functions are mediated by the tail domains, via binding to CD91 and calreticulin, and anti-inflammatory by the head domains, via binding to SIRP-1α [21]. SP-D has a fairly complex quaternary structure in which monomers (43 kDa) are assembled into tetramers of trimers thus forming dodecamers. Dodecamers may be further assembled into large multimeric structures (Figure 1A). Different from SP-A, the oligomerization of SP-D results in the burial of the tail domains while the head domains are exposed. Oligomerization is dependent upon the cysteine residues at positions 15 and 20 within the N-terminal tail region [22]. Mutation of these residues results in SP-D being incapable of forming any quaternary complex larger than a trimer [23]. Transgenic animals that are *SP-D* ^–/–^ and express a mutant form of SP-D with serine replacing the cysteines at positions 15 and 20 [24], still demonstrate enlarged and foamy macrophages within the airways. Furthermore, overexpression of the mutant protein in *SP-D* ^+/+^ results in the appearance of activated macrophages in the airspace. These observations strongly imply that oligomerization of SP-D is critical to its control of the inflammatory response of the lung.

**Figure 1:**
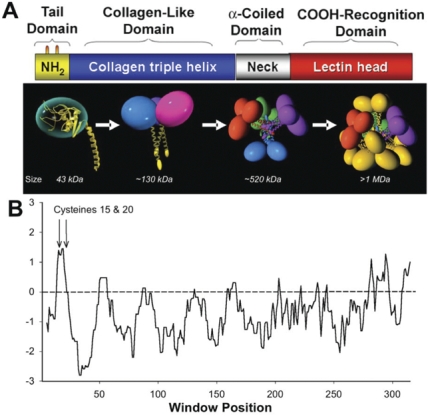
SP-D Structure (A) A model of SP-D structure. (Upper panel) The SP-D monomer (43 kDa) consists of a carbohydrate recognition domain which forms the globular head structure. This domain is connected to the collagen-like helical tail domain by a short, 30–amino acid, neck domain. At the end of the tail domain is the amino terminus in which cysteines 15 and 20 are positioned (shown as yellow projections). (Lower panel) Stylized representation of SP-D multimer assembly (note tail domains are shown shortened for ease of visualization). The head and neck domains drive the aggregation of the SP-D monomer to form a trimer of ∼130 kDa. These trimers associate to form a dodecamer (∼520 kDa). The forces holding this dodecamer together are unclear, although there is a dependency upon the amino-terminal cysteines as mutant lacking these cysteines do not form dodecamers. These dodecamers can assemble to a multimer of greater than 1 MDa. It is unclear whether the dodecamer is an essential intermediate in multimer formation. It should be noted that neither the trimer nor the dodecamer are globular proteins, due to the presence of the long collagen tail and thus under native conditions will behave as molecules with greater molecular radius. (B) Hydropathy plot of SP-D. A Kyle Doolittle hydropathy plot for the SP-D sequence was constructed using a window size of nine residues. A positive value indicates a region of hydrophobicity. The position of cysteines 15 and 20 are marked within the tail domain. As one can see, this is the most hydrophobic portion of the molecule.

Cysteines 15 and 20 are located within the most hydrophobic region of the SP-D protein (Figure 1B) [22]. The positioning of a reduced cysteine within such a hydrophobic region has been proposed as a motif for the formation of a SNO [13]. We propose that SP-D is a target for NO reactivity and that such chemistry may alter the oligomeric structure of SP-D and thus regulate its inflammatory function. We examined the forces involved in maintaining the dodecameric structure of SP-D. Furthermore, we have found that SP-D is *S*-nitrosylated by NO both in vivo and in vitro and that this modification is associated with alterations in quaternary structure. Furthermore, we show that nitrosylation results in a functional switch, activating the pro-inflammatory capabilities of SP-D. Thus, NO-mediated modification may explain the dichotomous nature of SP-D within innate lung immunity.

## Results

The oligomerization of SP-D into its dodecameric form is dependent upon the cysteines 15 and 20 in the hydrophobic tail of the monomer. This has led to the suggestion that the dodecamer may be held together via a series of disulfide bonds between the cysteines. To investigate this possibility, we chose to examine the reductive state of these cysteines via reaction with the alkylating agent N-ethyl maleimide (NEM). As shown in Figure 2A, we were able to label rat recombinant SP-D (rSP-D) with NEM linked biotin; however, we were unable to label rSP-D in which the cysteines 15 and 20 had been mutated to serine (ser 15/20 SP-D). These data indicate that within the recombinant protein, these two cysteines exist at least partially within a reduced state, but that the other cysteines located in the head domain are oxidized. Examination of these two proteins by SDS-PAGE further demonstrates the nature and importance of these cysteines (Figure 2B). With rSP-D, SDS-PAGE in the absence of thiol reductants results in a single band at the molecular size equivalent to a trimer. Treatment with either dithiothreitol or β-mercaptoethanol reduced rSP-D to a monomer. Interestingly ser15/20 SP-D was a monomer in SDS-PAGE analysis irrespective of thiol reduction. These data indicate that these two cysteines are critical in SP-D structure and that disulfide bonds involving them are critical in the trimeric structure but not in the dodecamer. Indeed, few inter-trimeric disulfide bonds exist in rSP-D, as shown by the single predominant band seen in nonreducing electrophoresis.

**Figure 2:**
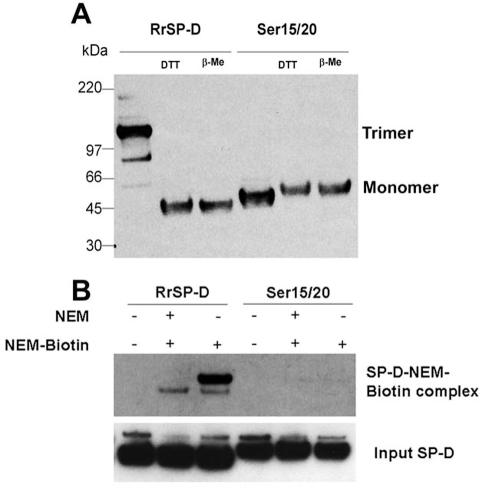
Role of Cysteine Residues 15 and 20 in Formation of SP-D Trimers (A) Recombinant rat SP-D (RrSP-D) or a mutant in which cysteines 15 and 20 have been mutated to serine (Ser15/20) were denatured under reducing (using mercaptoehtanol or dithiothreitol as the reductant) and non-reducing conditions. The resultant proteins were analyzed by SDS-PAGE and Western blotting with SP-D antibody. (B) RrSP-D and Ser15/20 were pre-incubated with NEM-linked to biotin at 37 °C for half an hour either with or without prior incubation with unlinked NEM. Biotin-labeling was determined by Western blotting following SDS-PAGE with anti-biotin antibody.

One of the proposed motifs for the formation of SNO is the presence of a cysteine residue within a hydrophobic pocket of protein structure [18,25]. Having established that these two cysteines were critically important to the dodecameric structure of SP-D and that this is not through the formation of disulfide bonds, we investigated whether they were targets for *S*-nitrosylation. We examined both bronchoalveolar lavage (BAL) and rSP-D following transnitrosation with *S*-nitrosocysteine (SNOC) (Figure 3A and 3B). As evidenced by the biotin-switch assay [16], SP-D, both within BAL and in recombinant form, was readily *S*-nitrosylated by SNOC. Analysis of BAL by native electrophoresis demonstrates that the multimeric/dodecameric forms are so large that they can barely enter the gel. However, following in vitro *S*-nitrosylation of BAL from mice overexpressing SP-D or rSP-D, the size of the SP-D complex is reduced such that lower molecular weight forms are visible (Figure 3A). In parallel, we analyzed BAL from mice overexpressing SP-D by gel filtration revealing (Figure 3B) that native SP-D exists in a complex ≥1 MDa in size (corresponding to the either the dodecamer or higher-order multimers, Figure 1A). Treatment of this BAL with SNOC reduces the complex size to below 720 kDa (corresponding to the trimer, Figure 1A). Lower–molecular weight SP-D (∼120 and 50 kDa, monomer and potentially dimer, respectively) is visible with and without SNOC treatment. Further native electrophoresis of the gel filtration fractions reveals that the >1-MDa fraction does consist of complexes that are too large to enter the gel, such as multimers and dodecamers [26,27], while the trimers are found in the 720-kDa fraction. The lower two fractions contain smaller forms of SP-D which may be an artifact resulting from the use of overexpressing mice. In summary, it appears that in vitro transnitrosation results in the formation of SNO-SP-D and alters the quaternary structure of SP-D such that its multimeric size is reduced.

**Figure 3:**
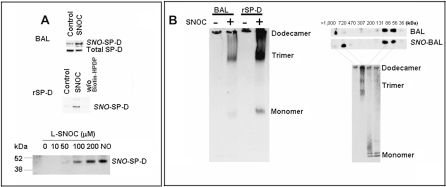
SNO-SP-D Formation In Vitro and Its Effect on Multimerization (A) (*Top*) SNO-SP-D formation in BAL. BAL from normal rats either with or without treatment with L-SNOC (200 μM) was analyzed for SNO-SP-D content by biotin-switch assay. Total SP-D content was also measured by immunoblot. (*Middle*) Transnitrosation of recombinant SP-D. Control and SNOC treated recombinant SP-D (0.2 μM) were analyzed by biotin-wwitch assay. W/o biotin-HPDP represents the assay performed in the absence of biotin linked [N-(6-biotinamido)hexyl-1′-(2' pyridyldithio) propionamide]. (*Bottom*) Recombinant SP-D (0.2 μM) was transnitrosated with increasing doses of L-SNOC or exposed to 200 μM authentic NO and then analyzed by biotin-switch assay. (B) SNOC treatment alters the conformational state of SP-D. (Left panel) BAL from SP-D overexpressing mice or 0.2 μM recombinant SP-D were treated with L-SNOC and subjected to native electrophoresis and Western blot for SP-D, revealing disruption of the native multimers to dodecamers and trimers. (*Upper right panel*) The BAL samples in the left panel were subjected to gel-filtration. Total protein from BAL (0.75 mg) in a volume of 250 μl was resolved onto a Superdex 200 HR 10/30 column (GE Healthcare Bio-Sciences) for size-exclusion chromatography (SEC) analysis. Protein extracts were resolved at flow rate of 0.3 ml/min in 25 mM HEPES, PH 7.25, and 150 mM NaCl using an Agilent 1100 Series HLC system. Fractions (0.5 ml) were collected and concentrated with 5000 NMWL Ultrafree-MC filters (Millipore). The gel filtration column was calibrated using the following mixture of globular proteins standards: thyroglobulin (669 kDa), ferritin (440 kDa), catalase (232 kDa), aldlase (158 kDa), albumin (67kDa), ovalbumin (43 kDa), chymotrypsin (25 kDa), and ribonuclease A (13.7 kDa). The void volume was determined from the elution migration of blue dextran (2,000 kDa). The fractions were analyzed by SDS-PAGE and Western blot for total SP-D content. The large multimers seen in control BAL are reduced in size upon SNOC treatment. (*Lower right panel*) Gel filtration samples containing SP-D were analyzed by native electrophoresis and Western blot, revealing that the 720-kDa fraction that arises upon SNOC treatment contains dodecamers and trimers of SP-D.

Previously it has been suggested, in particular for SP-A, that differential binding of the head and tail regions of the collectins by receptors on macrophages may control their anti- and pro-inflammatory functions [21,27]. In the structural model of SP-D (Figure 1A), it is proposed that within the multimer, the tail domains are buried and the head domains exposed; however, disruption of this structure, such as that shown with SNOC treatment (Figure 3), leads to exposure of the tail region. Therefore, it is reasonable to suggest that disruption of the multimeric structure by *S*-nitrosylation would lead to activation of those functions of SP-D mediated by the tail domain of the protein. We examined the ability of both modified and unmodified SP-D to act as a chemoattractant for macrophages. By using a modified Boyden chamber with RAW 264.7 cells as the target, BAL and rSP-D, both before and after treatment with SNOC, were assessed for their ability to induce chemotaxis (Figure 4). Both SNO-BAL and SNO-SP-D induced significantly greater RAW cell chemotaxis than their respective controls; furthermore, there was clearly a dose dependence to this induction (Figure 4A and 4B). This chemoattractant function of SNO-SP-D did not result from NO release and activation of guanylate cyclase, because the classical inhibitor ODQ, did not reduce the effect of SNO-SP-D (unpublished data). Because there are multiple potential cysteine containing proteins within BAL that could be transnitrosated by SNOC, one cannot from these data infer that the effect within BAL depends on the formation of SNO-SP-D. Therefore, the effect of SP-D immunoprecipitation upon SNO-BAL–induced chemotaxis was examined. Removal of SNO-SP-D from SNOC treated BAL ablates the chemotactic response mediated by this treatment (Figure 4C). The importance of SP-D in mediating the effects of SNO treatment upon chemotaxis was demonstrated by examining BAL from *SP-D* ^–/–^ mice. SNOC-treatment of *SP-D* ^–/–^ BAL failed to elicit an in increase in RAW cell chemotaxis (Figure 4D). Collectively, these data demonstrate that SNO-SP-D is responsible for the induction of macrophage chemotaxis following in vitro SNOC treatment.

**Figure 4:**
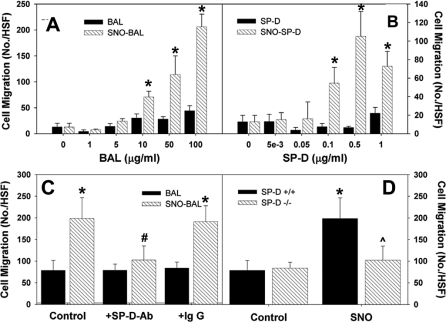
SNO-SP-D Induces Macrophage Chemotaxis (A and B) BAL and recombinant SP-D were assessed for their ability to induce RAW 264.7 macrophage migration using a modified Boydin chamber, following treatment with 200 μM SNOC or cysteine. (C) Using a BAL does of 100 μg/ml, cell migration was assayed subsequent to treatment with anti-SP-D or non-immune IgG. (D) SNOC treatment of BAL from *SP-D* ^–/–^ mice did not induce macrophage chemotaxis. (asterisk represents significantly different from BAL; pound symbol represents significantly different from control or IgG; carat symbol represents significantly different from SNO *SP-D* ^+/+^; *p* < 0.05)

SNOC treatment alters the quaternary structure of SP-D and results in the production of SNO-SP-D. NEM is only capable of alkylating residues 15 and/or 20 (Figure 2) suggesting that one or both of these residues would be the target for transnitrosation by SNOC. To confirm this supposition, we examined the ability of SNOC to transnitrosate both rSP-D and ser 15/20 SP-D (Figure 5A). rSP-D but not ser 15/20 SP-D was effectively nitrosated by SNOC as determined by the biotin-switch assay. In conjunction with these assays, the ability of the treated SP-D molecules to induce macrophage chemotaxis was also measured. SNO-rSP-D was found to be a potent inducer of chemotaxis, however, neither cysteine-treated rSP-D nor ser 15/20 SP-D had a significant effect (Figure 5B). This is despite the fact that within these molecules, the tail region is exposed, implying that nitrosation not only results in structural disruption but is also critical to the signaling function within macrophages.

**Figure 5:**
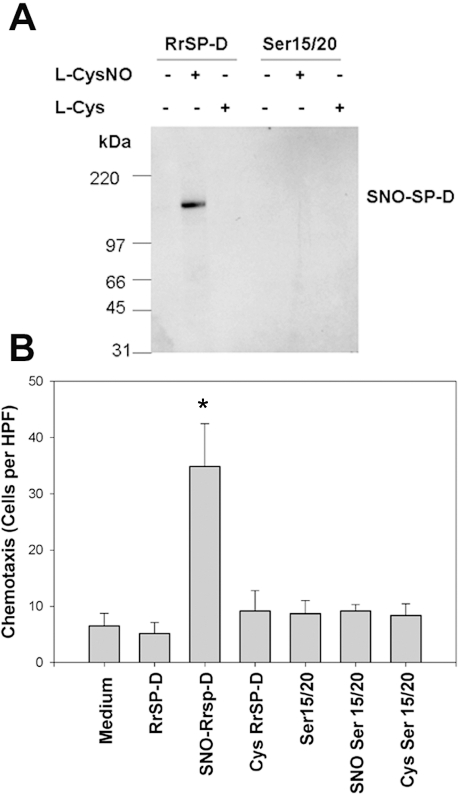
Chemotactic Activity of SNO-SP-D Trimers (A) Recombinant rat SP-D (RrSP-D) or the mutant (Ser15/20) was incubated with 200 μM l-cysteine or l-CysNO for 30 min at room temperature. SNO content was analyzed by Western blotting of non-reduced SDS-PAGE samples following use of the biotin switch assay. (B) Chemotactic activity recombinant and modified SP-Ds. 0.1 μg/ml of the treated SP-Ds in (A) were used to measure chemotaxis in a modified Boyden chamber assay with RAW 264.7 cells. Data are mean ± SEM. (asterisk represents significantly different from RrSP-D; *p* < 0.05)

Having shown that SP-D is a target for transnitrosation and that SNO-SP-D formed in this way has potential functional consequences, it remained to be demonstrated that this modification occurred in vivo. Previously, we used intratracheal administration of bleomycin as a model of acute lung injury within both rats and mice [28–30]. In rats, the peak inflammatory response is observed 7 d after injury. This inflammatory response resolves by day 14 and resultant fibrosis occurs around day 21. Within mice, this timeline is slightly extended with the inflammatory response extending out beyond 14 d. We examined the BAL fluid from control, saline-treated, and bleomycin-treated rats and mice for evidence of SP-D nitrosylation over the 21 d following treatment. In control and saline-treated rats, SNO-SP-D was either undetected or seen in marginal quantities; however, SNO-SP-D was readily detected following bleomycin-treatment peaking at day 2 and day 4 after bleomycin injury (Figure 6A). Therefore, SNO-SP-D formation precedes the peak inflammatory response, suggesting that nitrosylation may be an early event. Similar results were obtained in BAL samples from mice where SNO-SP-D was detected 8 d after bleomycin treatment but not in control or saline-treated animals (Figure 6B). To determine whether the quaternary structure of SP-D was altered with injury, we examined SP-D from injured and control rats at day 4 by Western blot following native and reducing gel electrophoresis. In native gels of control samples, none of the lower–molecular weight forms of SP-D was observed (Figure 6C). However, BAL from injured rats, examined in the same manner, clearly demonstrated lower–molecular weight forms of SP-D (Figure 6C). The presence of a small amount of lower–molecular weight SP-D in the saline-treated animals is not surprising, because this treatment has been shown to be injurious and elicit a slight inflammatory response [28]. These observations show that SNO-SP-D is formed both in vivo as well as in vitro and that this formation is associated with an altered conformational state for SP-D.

**Figure 6:**
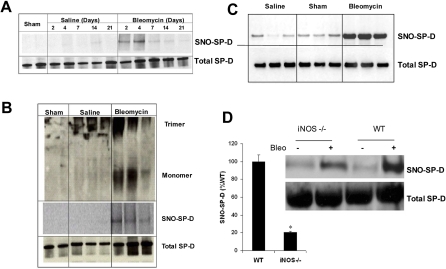
Acute Lung Injury in the Rodent Produces Results in SNO-SP-D Formation and Multimer Disruption (A) Untreated (control), saline, or bleomycin was intratracheally administered to Sprague-Dawley rats. BAL was collected at days 2, 4, 7, 14, and 21 after injection. BAL (upper) was subjected to biotin-switch assay to detect SNO-SP-D. (Lower) Total SP-D in BAL was identified by immunoblot. (B) Untreated (control), saline, or bleomycin was intratracheally injected at a dose of 8 U/kg in Sprague-Dawley rats. BAL was collected at day 4 after injection. BAL (upper) was subjected to electrophoresis for native gel to detect different fragments of SP-D. BAL (middle) was subjected to biotin-switch assay to detect SNO-SP-D. (Lower) Total SP-D in BAL was identified by immunoblot. (C) Untreated (control), saline, or bleomycin was administered to C57/BL6 mice intratracheally at a dose of 3 U/kg. BAL was collected at day 8 after treatment; total SNO content within the BAL was 0.2 ± 0.22 μM in control mice and 2.1 ± 0.88 μM in bleomycin treated. SNO-SP-D content was assessed by the biotin switch method. (D) SNO-SP-D in the BAL of bleomycin-treated wild type versus bleomycin-treated *iNOS* ^–/–^ mice (8-d post-injury) was measured by biotin-switch assay; Total input of SP-D was same between groups. (asterisk represents significantly different from wild type; *p* < 0.05). Representative blots show SNO-SP-D and total SP-D. Data are mean ± SEM.

To demonstrate the dependence of SNO-SP-D formation upon NOS-generated NO within this in vivo model, we examined SNO-SP-D generation in *iNOS* ^–/–^ mice. Previously, it has been demonstrated that inhibition of iNOS function, either by genetic or pharmacological means, results in a degree of resistance to bleomycin injury [31]. In addition SP-D has been shown to play a critical role in bleomycin induced acute lung injury [30]. We examined the BAL of both wild-type and *iNOS* ^–/–^ mice 8 d after bleomycin injury (Figure 6D). SNO-SP-D formation was reduced by over 80% in *iNOS* ^–/–^ mice relative to wild-type animals following injury. It is interesting that in both strains, SNO-SP-D formation was observed, suggesting that NO production is still increased within the *iNOS* ^–/–^ mice in this acute injury model.

The functional effects of SNO-SP-D within this model were examined by using the chemotaxis assay. As predicted, BAL from bleomycin-treated rats promoted significantly greater chemotaxis than that from saline-treated animals (Figure 7). Pretreatment of the BAL with ascorbate, which removes the NO moiety from SNO (Figure7A, inset), significantly reduced the chemotactic efficacy of BAL from bleomycin treated rats (Figure 7A). There was no effect of this treatment upon BAL-induced chemotaxis from saline treated rats. To confirm the importance of SP-D in the SNO-mediated increase, the chemotaxis assay was repeated using BAL that had been pre-immunoprecipitated with either antibodies to SP-D or non-immune IgG (Figure 7B). Neither antibody treatment had an effect on chemotaxis induced by BAL from saline-treated rats. However, anti-SP-D treatment produced a significant reduction in chemotaxis induced by BAL from bleomycin-treated rats, confirming the requirement for SP-D in the SNO-mediated effect. A word of caution is worthy, as one cannot confirm the effects of SP-D immunoprecipitation upon other chemotactic factors, such as cytokines, in these experiments; and thus the entire reduction observed may not result from SP-D removal. Although it is of note that non-immune IgG had no effect. These results confirm our in vitro observations in an in vivo model, namely that SP-D is nitrosylated, reduced in multimeric size, and becomes a pro-inflammatory signal.

**Figure 7:**
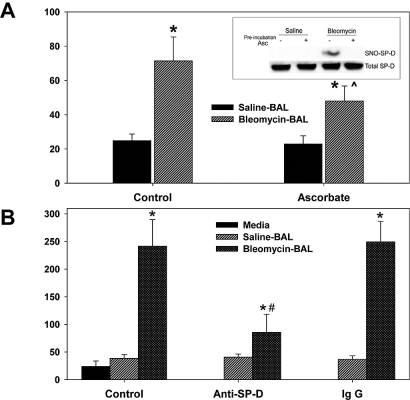
BAL from Bleomycin-Treated Rats Induces Macrophage Chemotaxis in Part through SNO-SP-D (A) BAL from bleomycin- and saline-treated rats were analyzed for their ability to induce RAW cell chemotaxis. Bleomycin BAL induced chemotaxis to a greater extent than saline BAL; however, pretreatment with ascorbate to remove SNO abrogated this response. The effect of ascorbate treatment on BAL SNO-SP-D content is shown in the inset where samples were analyzed by biotin-switch. (B) The importance of SP-D in this SNO-mediated increase is demonstrated by effect of SP-D immunoprecipitation. Bleomycin and saline BAL were analyzed for chemotactic effect following pretreatment with anti-SP-D or non-immune IgG. Only anti-SP-D pre-treatement reduced the increase in chemotaxis induced following bleomycin administration. (asterisk represents significantly different from saline-BAL; # represents significantly different from control or IgG; *p* < 0.05)

The observation of SNO-SP-D–mediated chemotaxis shows that there is a functional effect of nitrosylation but does not provide a mechanism for this effect. One of the main events that occurs during the induction of chemotaxis within a macrophage is the influx of extracellular calcium. To examine whether SNO-SP-D had an effect on intracellular calcium levels, RAW cells were loaded with the calcium responsive fluorescent dye, Fura-2. Control BAL had little effect upon the intracellular calcium concentration (Figure 8A), as there was no increase in fluorescence upon addition. However, SNO-BAL resulted in a significant calcium influx with a peak response approximately 2 min after its addition to the medium. Similar results were obtained with rSP-D, although the time constant of induction was much lower, as by 100 s, intracellular calcium levels had returned to normal. In addition to inducing chemotaxis, a predicted downstream consequence of such a calcium influx is the phosphorylation and activation of p38 MAPK. Figure 8B shows that SNO-BAL from wild-type mice, but not control BAL or SNO-BAL from *SP-D* ^–/–^ mice, was capable of inducing phosphorylation of p38 MAPK. These data establish that SNO-SP-D, but not native SP-D, is capable of inducing inflammatory signaling within macrophages and provides some insight into the mechanism of this signaling.

**Figure 8:**
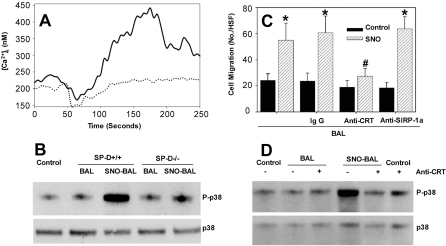
SNO-BAL Induces Inflammatory Signaling in RAW Cells via CRT (A) RAW 264.7 macrophages grown on coverslips were preloaded with fura-2AM and stimulated with BAL (100 μg/ml) or SNO-BAL (100 μg/ml) from SP-D^+/+^ mice. Change in [Ca^2+^]_i_ was record from 50–70 cells. (B) RAW 264.7 macrophages were treated with BAL or SNO-BAL from either *SP-D* ^+/+^ or *SP-D* ^–/–^ mice for 5 min. Cell lysates were analyzed for total p38 MAPK and its phosphorylated form (P-p38) by SDS-PAGE and Western blotted with phospho-specific primary antibody. (C) RAW 264.7 cells were pretreated with 2 μg/ml of anti-SIRP-1α, anti-CRT or an isotype control for 20 min prior to addition to the Boyden chamber. Treated cells were analyzed for chemotaxis to BAL (control) or SNO-BAL (SNO). (D) RAW 264.7 cell were pretreated with anti-CRT for 20 min prior to stimulation with BAL or SNO-BAL for an additional 20 min. Cell lysates were then prepared and analyzed for p38 phosphorylation via SDS PAGE and Western blot with phospho-specific primary antibody. (asterisk represents significantly different from control; pound symbol represents significantly different from control, IgG, or anti-SIRP-1α (*p* < 0.05)

Gardai et al. proposed that collectins exert their anti-inflammatory function through SIRP-1α and their inflammatory function through calretuclin (CRT)/CD91 [21]. It was proposed that the pro-inflammatory actions of SNO-SP-D, namely increased chemotaxis and intracellular signaling, occurred via binding to calreticulin. To investigate this possibility, the effects of pretreament of RAW cells with antibodies to CRT or SIRP-1α upon SNO-BAL-mediated chemotaxis were investigated. Figure 8C demonstrates that SNO treatment once again induced chemotaxis in RAW cells. Furthermore, pretreatment of the RAW cells with either non-immune IgG or anti-SIRP-1α had no effect upon the degree of chemotaxis in eithe rnormal or SNO-trated BAL. However, pretreatment with anti-CRT specifically reduced the SNO-BAL mediated chemotaxis to the level of untreated BAL. Pretreatment of RAW cells with anti-CRT resulted in a minimal increase in p38 phosphorylation (Figure 8D). However, while anti-CRT treatment had little effect on p38 phosphorylation within BAL-treated cells; it resulted in a significant decrease in SNO-BAL–induced phosphorylation. These results indicate that not only does nitrosylation of BAL, and hence SP-D, produce increased inflammatory signaling, as demonstrated by increased RAW cell chemotaxis and p38 phosphorylation, but that this activity is mediated by CRT.

## Discussion

The results presented here demonstrate that SP-D can be nitrosylated both in vitro and in vivo. Further, they demonstrate that this nitrosylation is associated with disruption of the multimeric structure of native SP-D and that these modifications result in a pro-inflammatory signaling activity with macrophages. From these observations, we propose an extension of the model of Gardai et al. specifically for SP-D [21] (Figure 9). Within this model, SP-D exists within the lung lining fluid in large multimers, such that only the head domain, the carbohydrate recognition domain, is exposed. These multimers are capable of interacting either with invading pathogens or to the cell surface receptor, SIRP-1α. SP-D binding to SIRP-1α results in suppression of p38 phosphorylation via SHP-1. Thus multimeric/dodecameric SP-D could reduce inflammatory signaling via SHP-1–mediated inhibition of p38 and reduced activation of NF-κB. Presumably the interaction of the head domain with pathogenic molecules, such as lipopolysaccharide (LPS), results in SP-D subunits, probably trimers, being pulled from the multimer resulting in tail domain exposure. Exposed tail domains are then available for interaction with CRT/CD91, resulting in activation of p38 phosphorylation and potentially NF-κB and its downstream inflammatory consequences. It is important to note that the data here do not address NF-κB activation, and this part of the model remains a proposal at this time.

**Figure 9:**
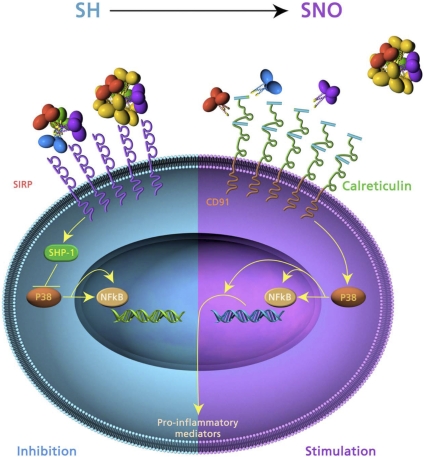
A Model of the Pro- and Anti-Inflammatory Functions of SP-D Under non-inflammatory conditions, SP-D remains in large multimeric or dodecameric forms in which the tail domains remain buried. The head domains bind to SIRP-1α and activate the kindase SHP-1. SHP-1 activation inhibits p38 activations, potentially resulting in the blockaged of NF-κB action and the inhibition of infammatory function. Under inflammatory conditions the production of NO leads to the formation of SNO-SP-D and the disruption of the multimeric structure. The tail domains now become exposed and bind to calreticulin. This results in p38 phosphorylation via CD91, potentially leading to NF-κB activation and the production of pro-inflammatory mediators. Presumably other actions which result in disruption of SP-D multimeric structure may also be pro-inflammatory. (Image credit: Kirk Moldoff)

NO can be produced throughout the lung either from nNOS, which is present throughout the pulmonary epithelium [32], or from iNOS, whose synthesis is induced in either pulmonary epithelial cells or alveolar macrophages by pro-inflammatory cytokines [33]. The presence of two reactive cysteines within the hydrophobic tail domain (Figure 1) makes SP-D a prime target for NO-mediated post-translational modification, resulting in the formation of SNO-SP-D. As a consequence of this nitrosylation the multimeric structure of SP-D is disrupted and trimers are released. The resulting exposure of the tail domains allows for interaction with CRT/CD91 and the activation of p38 phosphorylation and potentially downstream pro-inflammatory signaling. In this way, pro-inflammatory signaling of SP-D can be induced by either the presence of pathogens or chemically via NO production. Previously it has been demonstrated in hemoglobin that changes in protein structure can affect the ability of cysteine residues to be nitrosylated or not [34]. In addition, the functions of a wide range of proteins—including channel proteins such as CFTR, G proteins, metabolic enzymes, and transcription factors—have been shown to be regulated by SNO formation both in vivo and in vitro [13,35]. However, although SNO modification has been reported to induce assembly in both dynamin and arginase 1 [36,37], this is the first demonstration, to our knowledge, of a SNO-modification promoting multimeric disassembly and a switch in function, and thus represents a novel mechanism of action for this redox-based signal.

Our model provides a mechanistic basis to explain the apparently dichotomous nature of SP-D as both a pro and anti-inflammatory molecule [19,27,38]. The key to this model is that it is the exposure of the tail domains that allows for CRT binding [21]. We have not at this time determined whether the association of SNO-SP-D with CRT requires the presence of the NO group or if it is merely the tail domain exposure that is required. In the later instance, one can see that other chemical modifications could also result in pro-inflammatory SP-D signaling. For instance, primary oxidation of these same cysteine residues would result in sulfenic acid formation, which can reasonably be predicted to also cause multimer disassembly. In regard to this question, recombinant SP-D, which is often in the trimeric state, can induce neutrophil chemotaxis; although it should be noted that in the studies presented here recombinant SP-D had a minimal chemotactic effect upon RAW cells. Also the ser 15/20 mutant, in which the tail domains are exposed, does not induce chemotaxis implying that there is a greater level of complexity to this signaling.

In the original model of Gardai et al. [21], which was primarily based upon studies with SP-A, the association of the collectin with a pathogen was the required step to initiate CRT binding. It was proposed that the tail domains had a low affinity for CRT and thus the aggregation of collectin molecules upon a pathogen was required to provide sufficient binding energy. Within our model, one would predict that SNO-SP-D is not required to be associated with any other macromolecule in order to bind CRT, as CRT-mediated effects can be produced by SNO-SP-D alone (Figure 8). Therefore, it is implied within this model that the tail domain of SP-D has a higher affinity for CRT than that of SP-A. This could result from the presence of the nitrosothiol moiety upon the modified SP-D or be simply a feature of the exposed SP-D tail domain. The precise mechanism of SP-D and CRT interaction warrants further investigation.

Examination of the BAL samples from the bleomycin-treated rats and mice (Figure 4) reveals that, although nitrosylation of SP-D occurs and that there is disruption of the multimeric structure, much of the SP-D is unaffected. Therefore, one can safely assume that SP-D is binding to both SIRP-1α and to CRT, and that the balance of this binding determines what is the final consequence of cellulare association with SP-D. In other words, SP-D may operate as an integrator of the status of the lung lining, initiating inflammatory responses under a variety of pathological conditions, such as infection or nitrosative stress, while maintaining a quiescent state in the absence of stress. It is important to note that SP-D is only one of a number of potential target proteins that can be nitrosylated within the airway, including channel proteins, G proteins, and transcription factors [35]. This is of particular relevance when one considers the effects of inhaled NO, whose effects could be either pro- or anti-inflammatory, depending upon the conditions of its administration and the subject's lung. For instance, in our model, SNO-SP-D would initiate a pro-inflammatory response via NF-κB activation; however, SNO formation on NF-κB itself has been shown to inhibit activation [39]. Therefore, one can see that the potential effects of NO inhalation on pulmonary inflammation will be condition dependent.

In summary, the studies outlined here reveal that SP-D function can be controlled through post-translational modification by NO; and this modification alters the quaternary structure of SP-D by disrupting its multimeric state such that trimers are formed. In addition, the formation of SNO-SP-D trimers initiates a pro-inflammatory response through CRT/CD91 and p38 activation. This is a novel mechanism of regulation for both NO and collectins and allows for the consideration of a novel mode of action for NO.

## Materials and Methods

### SNO-protein synthesis.

In order to generate *S*-nitrosylated proteins, BAL (300 μg/ml of total protein) or recombinant rat SP-D (10 μg/ml) was exposed to L-CysNO (200 μM) in the dark for 30 min at room temperature. The *S*-nitrosylated proteins were then purified on a G25 Sephadex column (Biorad). The generation of protein *S*-nitrosocysteine content was evaluated by reaction with copper/cysteine coupled with chemiluminescent detection, with *S*-nitrosoglutathione as the standard, [40] using a Sievers NOA 280 (GE). Within BAL, under these conditions 23.7 ± 6.91 nmoles of SNO per mg of protein (mean ± SD, *n* = 4) were generated from a basal level of 3.3 ± 0.96 nmoles/mg protein (mean ± SD, *n* = 4).

*Biotin-Switch Assay for Detection of SNO-SP-D.* Detection of SNO*-*SP-D was performed via an adaptation of the biotin switch method [16]. BAL (30 μg total protein) in HEN buffer (25 mM Hepes, pH 7.7/0.1 mM EDTA/0.01 mM neocuproine),and 20 μM *N-*ethylmaleimide (NEM) at 37 °C for 30 min to block free thiols. Excess NEM was removed by protein precipitation using cold acetone. Protein pellets were resuspended in HENS buffer (HEN 1% SDS), SNO bonds were decomposed by adding 20 mM sodium ascorbate. The newly formed thiols were then linked with the sulhydryl-specific biotinylating reagent *N-*[6-biotinamido)-hexyl]-1-(2-pyridyldithio) propionamide (Pierce). Biotinylated proteins were precipitated with Streptavidin-agarose beads and Western blot analysis was performed to detect the amount of SP-D remaining in the samples.

### Western blots.

Immunoblotting for SP-D was performed using an equal protein 5μg of BAL fluid per lane. Samples were loaded on a 4–12% precast NuPAGE Bis-Tris polyacrylamide gel (Invitrogen). Proteins were electrophoresed using MEPS-SDS buffer at 200 V for 35–45 min per the manufacturer's instructions (Invitrogen). Native gel electrophoresis was performed using a Novex 4% tris-glycine gel (Invitrogen). Samples were mixed with a cold native gel sample buffer (Invitrogen) before loading. Electrophoresis was run at room temperature at a constant voltage of 80 V for 5 h. Proteins were then transferred from the gel to a nitrocellulose membrane using the XCell II Mini-Cell and sandwich blot module (Invitrogen) in Bicine-10% methanol-0.01% SDS transfer buffer (Invitrogen) at 30 V for 1 h. Blots were blocked for 1 h at room temperature with 10% nonfat milk and then incubated with primary SP-D antibody (1:10,000 dilution, The SP-D antibody, a rabbit polyclonal antibody was raised against purified rat SP-D as described previously) overnight at 4 °C. Blots were then incubated with 1:5,000 goat anti-rabbit IgG-horseradish peroxidase for 2 h at room temperature. Signal was detected using the enhanced chemiluminescence kit (Amersham), and blots were exposed to Kodak Biomax MS film.

### Model of bleomycin injury.

All animals for this study were housed in the Animal Care Facility of The Children's Hospital of Philadelphia under standard conditions with free access to food and water. All animal experimental protocols were reviewed and approved by the Animal Care and Use Committees of both the Children's Hospital of Philadelphia and The University of Pennsylvania. Either human clinical-grade, sterile, and lipopolysaccharide (LPS)-negative saline or 8.0 U/kg of bleomycin sulfate (Bristol Myers Squibb) in 25 μl of saline was administered intratracheally to 6-wk-old (200–250 g) male Sprague-Dawley rat littermates (Charles River Breeding Laboratories) as previously described [29]. Similarly, 8-wk-old C57/B6 mice (25–35 g) were administered with either 50 μl of either LPS-negative saline or clinical grade, sterile, and LPS-free bleomycin sulfate (3.0 U/kg; Bristo Myers Squibb) as previously described [30].

### Chemotaxis assay.

Directed migration (chemotaxis) of cells was performed as previously described [25] Briefly, 50 μl of cells suspended at 2 × 10^6^ cells/ml in DMEM were placed in the upper wells of a 48-well microchemotaxis chamber (Neuro Probe). The lower chambers contained 41 μl of test solution, consisting of DMEM and either nothing (control); various concentrations of SP-D, SNO-SP-D, BAL, or SNO-BAL. All test solutions were used in triplicate in each assay. A polyvinylpyrrolidone-free polycarbonate filter was placed between the wells along with the rubber gasket of the assembly. The filters used for macrophage chemotaxis had 5-μm pores (Neuro Probe). The chamber was incubated at 37 °C with 5% CO_2_ for 3 h, and then disassembled. Nonmigrating cells were scraped from the upper surface, and the migrating cells were stained with the Hemacolor differential blood stain. The filter was placed on a glass coverslip and mounted with immersion oil onto a glass slide. Cells that migrated through the filter were counted in ten randomly selected oil-immersion fields in each well at 1,000× magnifications. Data were expressed as cells per oil- immersion field for the three wells used for each solution.

### Measurement of [Ca^2+^]_i_.

RAW cells (2 × 10^6^) were plated on glass coverslips (Fisher Scientific) sized to fit a homeothermic perfusion chamber platform of an inverted Nikon microscope. The cells were loaded with 5μM fura-2 acetoxymethylester (Molecular Probes) and 0.2 mg/ml pluronic F-127 (Molecular Probes) in 2 ml HBSS supplemented with 1% FBS and 1.25 mM CaCl_2_ for 30 min at 37 °C. The cells were stimulated with HBSS at 37 °C containing BAL or SNO-BAL and excitation was performed at 334 and 380 nm with two narrow-bandpass filters. The emitted fluorescence was filtered (520 nm), captured with a Hamamatso CCD video camera (512 × 480-pixel resolution), digitized (256 gray levels), and analyzed with SimplePCI (Version 3.7.9) software. The amount of Ca^2+^ was calculated by comparing the ratio of fluorescence at each pixel to an in vitro 2-point calibration curve. The Ca^2+^ concentration presented was obtained by averaging the values of all pixels over a cell body. The data points were collected at intervals of 5 s.

### p38 MAPK analysis.

RAW cells (1 × 10^6^ cells./ml) were cultured overnight, then incudbated with Bal or SNO-BAL (100 μg/ml) for 10 min. If anti-caltericulin was used in the experiments, the antibody (2 μg/ml) was added 30 min before the stimulation. Cells were lysed in lysis buffer [Hepes 20 mM, NaCl 150mM, Glycerol 10%, Triton X100 1%, EGTA 1mM, MgCl_2_ 1.5mM, pH = 7.4] containing protease inhibitors (PMSF 1mM, NaPyrophosphate 10mM, NaF 50mM, Na Orthovanadate 2mM, Lactacystin 1μM, AEBSF 1mM, EDTA 0.5mM, Bestatin 65μM, E-64 0.7μM, Leupeptin 0.5μM, and Aprotinin 0.15μM), and resolved in 4–10% SDS-PAGE, and blotted to a PVDF membrane as outlined above. The membranes were probed with a phosphospecific antibody to p38. To confirm the equal loading, the membranes were stripped and reprobed for p38.

## Abbreviations

BAL: bronchoalveolar lavage
CRT: calretuclin
NEM: N-ethyl maleimide, NO, nitric oxide
rSP-D: recombinant SP-D
SP: surfactant protein
SNO: *S*-nitrosothiol
SNOC: *S*-nitrosocysteine
